# Assessment of the Presence of Partially Hydrogenated Oils (PHOs) as a Source of Industrially Produced Trans Fatty Acids (i-TFAs) in Packaged Foods in Poland, Pre- and Post-Implementation of EU Regulation 2019/649

**DOI:** 10.3390/nu17061057

**Published:** 2025-03-18

**Authors:** Edyta Jasińska-Melon, Hanna Mojska, Bogumiła Krygier, Sławomir Garboś

**Affiliations:** Department of Nutrition and Nutritive Value of Food, National Institute of Public Health NIH—National Research Institute, 24 Chocimska Street, 00-791 Warsaw, Poland; bkrygier@pzh.gov.pl (B.K.); sgarbos@pzh.gov.pl (S.G.)

**Keywords:** food, labelling, partially hydrogenated oils (PHOs), industrially produced trans fatty acids (i-TFAs), palm oil

## Abstract

**Background/Objectives:** Trans fatty acids (TFAs) are risk factors for cardiovascular diseases. TFAs are classified as natural (r-TFAs) or industrially produced (i-TFAs). The primary source of i-TFAs is partially hydrogenated oils (PHOs). The European Union implemented Commission Regulation 2019/649, setting a limit of i-TFAs in food. The World Health Organization (WHO) has emphasised the need to completely eliminate PHOs from global food supplies. This study aimed to assess the content of PHOs in food, based on the declared ingredient composition on product labelling, pre- and post-implementation of Regulation 2019/649. The types of fats used as PHOs substitutes were also assessed. **Methods:** The study material consisted of product labels produced before (*n* = 1224) and after (*n* = 779) the implementation of Regulation 2019/649. An analysis of the fats declared in the ingredient lists of these products was carried out, together with an evaluation of the PHOs substitutes used. **Results:** Before the entry into force of Regulation 2019/649, 6.9% of the 1224 products evaluated contained PHOs. After the implementation of the Regulation, PHOs were not listed on the label of any of the 779 products evaluated. Among the 84 products that contained PHOs before Regulation 2019/649 came into force, 36 were no longer available on the market. The remaining 48 used palm oil as the primary PHOs substitute. **Conclusions:** The introduction of legal limits for i-TFAs in foods appears to be an effective strategy for reducing the dietary intake of TFAs. The commonly used PHOs substitute is palm oil with significant amounts of saturated fatty acids.

## 1. Introduction

The trans isomers of unsaturated fatty acids (trans fats, TFAs) present in food originate from both natural (r-TFAs) and industrial (i-TFAs) sources. The natural source of r-TFAs in the diet is the milk and meat of ruminants. In the forestomach (rumen) of these animals, the biohydrogenation and conversion of unsaturated fatty acids present in the feed to TFAs take place under the influence of the bacteria present there (*Butyrivibrio fibrisolens*). The maximum content of r-TFAs in milk and dairy products, as well as in meat and its processed products, does not exceed 6–8% of the total fatty acids. Trans isomers of oleic acid 18:1 (18:1 t) dominate, with the Δ11 18:1 t isomer, known as vaccenic acid, being the most prevalent among positional isomers. It is also important to emphasise that the content of r-TFAs remains relatively constant within specific ranges and is characteristic of the type of animal fat. Sources of industrially produced TFAs are mainly hydrogenated oils/fats (PHOs), usually vegetable and products made from them. According to pan-European studies conducted in the 1990s (The TRANSFAIR Study) [1,2,3], the content of i-TFAs in partially hydrogenated fats exceeded 50% of all fatty acids. These studies found that confectionery products contained the highest levels of TFAs. Hard margarines (block margarines) contained approximately 30%, while frying and baking fats had TFAs content reaching up to 50% of the total fatty acids. Small amounts of i-TFAs (1–2% of total fatty acids) are also formed during the oil refining at the deodorisation step and during high-temperature (>200 °C) frying of vegetable fats. The amounts and types of i-TFAs formed during the industrial hydrogenation of vegetable oils are somewhat unpredictable; but, among the 18:1 t positional isomers, elaidic acid (Δ9 18:1 t) dominates, in contrast to animal fats, where vaccenic acid predominates. It is important to emphasise that no analytical method currently available can distinguish between TFAs of natural and industrial origin [4].

The use of partially hydrogenated fats in foods, including in the form of margarines and other spreads for breading or frying, as an alternative to animal fats, has a long history of more than 100 years. As early as the late 1950s, there were reports linking the consumption of TFAs present in partially hydrogenated fats, to an increased risk of heart diseases [5]. It is now widely recognised that TFAs in the diet are a known risk factor for cardiovascular diseases. Excessive intake of TFAs leads to an increase in total cholesterol (TC) and low-density lipoprotein (LDL) cholesterol concentrations in blood serum, as well as a decrease in high-density lipoprotein (HDL) cholesterol concentrations [6,7,8]. This contributes to the development of cardiovascular diseases, including atherosclerosis and coronary heart disease (CHD), which have been a global public health concern for many years. De Souza et al. [9] estimated that high TFAs intake increased the risk of death from all causes by 34%, death from CHD by 28% and the development of CHD by 21%. With a high intake of i-TFAs alone, a 30% increase in the risk of CHD development and an 18% increase in the risk of death from CHD were noted. In addition, more than half a million deaths from CAD worldwide each year can be attributed to “high TFAs diets,” defined as an intake of TFAs from all sources above 0.5% of dietary energy (>0.5%E) [10]. It should also be noted that high TFA intake is associated with impaired fertility [11], as well as an increased risk of obesity, diabetes [12], neurodegenerative diseases [13], cancers [14] and allergic diseases in infants and young children [15]. It is worth noting that TFAs can cross the placental barrier and the blood–breast milk barrier. Thus, it poses a risk to the developing foetus and breastfed infants. The presence of TFAs in breast milk has been reported in numerous studies worldwide [16]. Furthermore, an inverse correlation was found between the maternal serum, umbilical cord and vessel walls TFAs concentrations, and the long-chain polyunsaturated fatty acids (LC-PUFAs) concentration, primarily docosahexaenoic acid (C22:6 n-3, DHA) [17,18]. Therefore, the presence of TFAs in certain food products can be an indicator of their potential impact on human health, and thus, the associated risk of developing various diet-related diseases.

According to experts from the European Food Safety Authority (EFSA) [EFSA 2010], the intake of TFAs should be as low as is possible within the context of a nutritionally adequate diet (ALAP—as low as possible). In the European Union (EU), Commission Regulation (EU) 2019/649 [19] sets a maximum limit of 2 g of i-TFAs per 100 g of fat in food intended for the final consumer and food intended for retail sale. The World Health Organization (WHO) has also introduced a six-step comprehensive action plan, known as REPLACE. This plan aims to eliminate i-TFAs from global food supplies [20]. According to WHO experts, partially hydrogenated fats should be replaced with plant-based fats and oils, excluding tropical oils such as palm and coconut oils. Palm oil contains around 50% saturated fatty acids (SFAs) (a quantity similar to that of animal fats), and coconut oil can contain up to 90% SFAs. For this reason, these oils are not recommended for use in diets for all age groups [21].

Given the challenges associated with taking action and implementing effective measures to prevent diet-related diseases, the primary objective of this study was to assess how and to what extent food manufacturers have adapted to the requirements of current regulations aimed at reducing i-TFAs in foods. This objective was pursued through the following: 1/a comparative analysis of the content of partially hydrogenated oils (PHOs) as a source of i-TFA in food, based on the declared product composition on labels, both before and after the implementation of Commission Regulation (EU) 2019/649; 2/an evaluation of the types of fats used as PHOs substitutes.

## 2. Materials and Methods

### 2.1. Study Design

**Figure 1:**
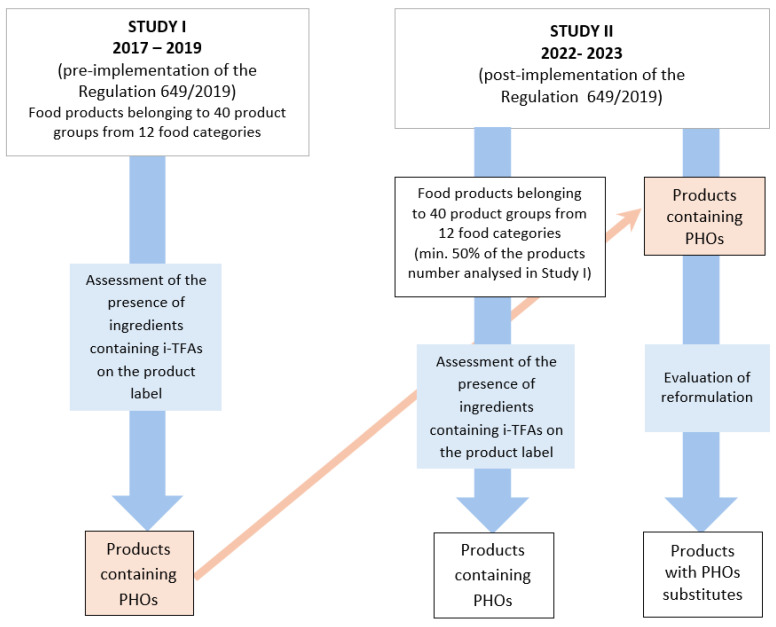
Research scheme. PHOs: partially hydrogenated oils, TFA: trans fatty acids.

The research scheme is shown in Figure 1.

Between 2017 and 2019 (Study I), the labels of 1224 food products available in retail sales on the Polish market were assessed using keywords identified in Table 1. The evaluation focused on identifying the type of fat used in the product, with particular attention given to the content of partially hydrogenated oils (PHOs) and tropical oils (palm oil and coconut oil). Product categories and groups were selected based on the current knowledge regarding trans fatty acid (TFAs) content in various food products. These data are available in the electronic database on TFAs content in food in Poland (https://izomery.pzh.gov.pl, accessed on 11 January 2025). The products were classified into 12 food categories and 40 defined product groups. Within each group, commonly available product types in retail outlets were selected. For each product type, all available brands/manufacturers of the product in the selected retail outlet were chosen for evaluation.

The product composition listed on the labels was analysed for the presence of partially hydrogenated oils (PHOs) and tropical oils (palm and coconut oils), regardless of their position in the ingredient list. Table 1 presents the keywords used to identify products containing PHOs and/or tropical oils. To identify products containing i-TFAs, only specific terms were used that unequivocally indicated the presence of these fatty acids in the ingredient list. Non-specific terms, such as “margarine” or “hydrogenated oil/fat,” which may or may not indicate the presence of i-TFAs, were not considered. Similarly, to identify products containing tropical oils, only explicit terms were used that clearly indicated their presence in the ingredient list, excluding broader terms such as “vegetable oils” or “plant-based fats.”

Based on the descriptions of partially hydrogenated oils/fats and tropical oils used by manufacturers in the ingredient lists of the assessed product labels, the following five product group descriptions were identified. These descriptions were subsequently utilised to characterise individual products and classify them into the appropriate groups:

- *Products containing partially hydrogenated oils/fats:* foods containing ingredients that are sources of i-TFAs (PHOs, including tropical oils such as partially hydrogenated palm and/or coconut oil).
- *Products containing tropical oils:* foods containing palm oil and/or coconut oil, excluding partially hydrogenated tropical oils such as palm and/or coconut oil.
- *Products containing partially hydrogenated oils/fats and tropical oils*: foods containing ingredients that are sources of i-TFAs (PHOs, including tropical oils such as partially hydrogenated palm and/or coconut oil), as well as foods containing palm oil and/or coconut oil.
- *Products containing hydrogenated oils/fats*: Foods containing hydrogenated oils/fats of unknown origin and degree of hydrogenation (fully/partially hydrogenated).
- *Products without partially hydrogenated oils/fats and tropical oils*: Foods that do not contain ingredients that are sources of i-TFAs, nor do they contain palm oil and/or coconut oil.

Between 2022 and 2023 (Study II), an evaluation was conducted to assess the effectiveness of the implementation of Commission Regulation (EU) 2019/649 in Poland, which sets the legal limit of i-TFAs in food (2 g per 100 g of fat). The study aimed to assess at least 50% of the products evaluated in Study I but were produced after the entry into force of the Regulation 2019/649. The selection of food products for the evaluation of the declared fat type on the labels was carried out within the same 12 food categories and 40 product groups identified in Study I. However, most of these were not products from the same brands or manufacturers as in Study I. Additionally, Study II included re-evaluation of the labels of those products from Study I that contained partially hydrogenated oils (PHOs) (Figure 1).

In Study I and Study II, product labels were assessed for items sourced from discount retailers (sales area: 300–1000 m^2^), supermarkets (sales area: 400–2499 m^2^) and hypermarkets (sales area: >2500 m^2^), as these are the primary food purchasing locations in Poland [22]. Based on data from a report on the Polish retail food trade market [23], 10 retail chains with the largest market share in Poland were selected. It was assumed that the products sold in the selected stores of these chains were identical to those sold in other stores of the same chains nationwide. The area of analysis for the food product labels was Warsaw, one of the biggest Polish towns and the Poland capital.

The data from the analysed food product labels were gathered by trained personnel and systematically recorded in an Excel database. Over 25,000 records for food products were compiled, including the following information: type of product, product brand, trade name, manufacturer name and address, date and location of analysis, batch numbers, production date, expiry date, composition (as stated on the product label), weight and/or volume of the packaging, fat content (per 100 g of product and per portion), saturated fatty acid content (per 100 g of product and per portion), portion-related information, and other relevant details specific to particular product groups, such as preparation instructions (e.g., for food concentrates) or target age group (e.g., for food for specific groups).

### 2.2. Statistical Analysis

The presence of selected fat ingredient designations in the ingredient lists provided on food product labels was analysed across two time periods at the product group and food category levels. The data obtained are presented as the number of products (N) and the percentage (%) of products containing the selected ingredients out of the total number of evaluated products. The calculated data represent precise values; therefore, confidence intervals were not provided. All results were processed and analysed using Microsoft Excel, available as part of the Microsoft Office 365 Suite.

## 3. Results

Table 2 presents the categories and product groups, with labels assessed in terms of the type of fat used, in both Study I and Study II, along with the number of products in each category.

As the data in Table 2 show, in both Study I and Study II, the largest group of products whose labels were assessed for information on the type of fat used consisted of food concentrates, followed by confectionery products and dairy products. Conversely, the lowest number of assessed labels were for fish products, vegetable products and fast food products.

Table 3 presents the results of the label assessment for 1224 food products available on the Polish market between 2017 and 2019 and produced before the entry into force of Commission Regulation (EU) 2019/649.

Among the 1224 assessed food products, 93.1% (*n* = 1140) did not contain ingredients that are sources of i-TFAs. Partially hydrogenated vegetable oils/fats were present in the composition of 84 products (6.9%). For 53 products in this group (4.4% of all the products assessed), this was the only type of fat used in production.

For the remaining 31 products (2.5% of all the products assessed), the manufacturer declared the presence of both partially hydrogenated oils/fats and tropical oils. Products containing PHOs were distributed, in decreasing order, across the following six food categories: food concentrates (*n* = 40), bakery products (*n* = 21), confectionery products (*n* = 18), fast food products (*n* = 3), snack products such as chips and crisps (*n* = 1) and meat products (*n* = 1).

Tropical oils (mainly palm oil) were present in the composition of a total of 575 products (47%). In this group, 544 products (44.4% of all the products assessed) listed tropical oils as the only type of vegetable fat used in production. Products containing tropical oils were found across 11 of the 12 food categories analysed. The highest number of products declaring the presence of tropical oils was in the food concentrates category (*n* = 229, 62%), followed by confectionery products (*n* = 157, 68%) and bakery products (*n* = 122, 82%) (Table 3).

Table 4 presents the results of the assessment of labels for 779 randomly selected products, produced after the entry into force of Commission Regulation (EU) 2019/649, conducted in 2022–2023. The analysis included products from the same categories and groups as those examined in 2017–2019 but not all products came from the same brands and manufacturers.

Of the 779 products analysed, none contained PHOs (Table 4). At the same time, nearly 40% of the products (*n* = 309; 39.7%) from six food categories (fast food products, food concentrates, confectionery products, bakery products, snack foods, dairy products) contained tropical oils, primarily palm oil. The category with the highest number of products containing tropical oils was food concentrates (*n* = 129; 64.8% of all products in this category). It should be underlined that 118 products in this category contained palm oil as a sole source of fat, 1 product contained coconut oil and the remaining 10 products contained both palm oil and coconut oil. In confectionery and bakery products, the presence of tropical oils was found in over 60% of the products from these categories, *n* = 83 (65.9%) and *n* = 55 (63.2%), respectively. The majority of these products contained palm oil. In contrast, in the categories of fast food products, snack products such as chips and crisps and dairy products, the presence of tropical oils (mainly palm oil) was found in 20% (snack products) to approximately 40% (fast food products) of the products analysed in these categories.

In the composition of 210 products (27% of all products analysed during this period) from the remaining six food categories, no tropical oils were found.

Furthermore, between 2022 and 2023 (Study II), the re-evaluation focused solely on products that contained PHOs prior to the implementation of Commission Regulation (EU) 2019/649 (Study I). The market analysis for re-identifying 84 products that contained PHOs in Study I revealed that, between 2022 and 2023, approximately 43% of the aforementioned products were no longer available in retail outlets. These products were likely discontinued. Only 48 (about 57%) of the 84 products initially containing PHOs in the 2017–2019 period were identified. The results are presented in Table 5.

These products belonged to five food categories, with the largest group still consisting of food concentrates (48% of all evaluated products). In the majority (52.1%) of the products assessed in Study II, PHOs were replaced exclusively with tropical oils, ranging from 30% to 100% depending on the food category. It should be noted that in one-third of these products, PHOs were replaced with vegetable oils other than tropical oils. In a few samples, the fat fraction reformulation involved replacing PHOs with animal fats, as well as a combination of tropical oils and animal fats.

## 4. Discussion

In our study, we compared the composition of retail products, focusing on the type of fat declared on the label, with particular emphasis on the presence of PHOs pre- and post-implementation of Commission Regulation (EU) 2019/649 [19]. This approach allowed us to assess the effectiveness of the legal requirements that set a maximum allowable content of i-TFAs at 2 g/100 g of fat in food intended for final consumers and in food intended for retail trade. According to our knowledge, this is the first study of its kind both in Poland and in other European countries.

Our study, conducted between 2017 and 2019, indicates that the presence of PHOs (a source of i-TFAs) was listed on the label of 6.9% of the products (*n* = 84) out of the total 1224 products whose labels were assessed prior to the implementation of Commission Regulation (EU) 2019/649. The highest percentage of such products was found in the categories of bakery products (14%), food concentrates (11%) and confectionery products (7%). Similar findings were obtained by Onacik-Gür et al. [24] in their studies conducted over 10 years ago, which analysed the labels of 2132 products in Poland. The authors of that study also identified food concentrates, confectionery products and bakery products as the main sources of PHOs. However, the percentage of products containing PHOs in their study was higher compared to our findings, at 44.1% for filled chocolates, 29.5% for bakery products and 27.1% for instant soups. In their study, the overall percentage of products containing PHOs was 28.1%, which was more than four times higher than in our study. The lower percentage of products containing PHOs in our study, compared to Onacik-Gür et al. [24], suggests that producers had already started taking voluntary actions to limit the use of PHOs in food products, even before the legal requirements came into force.

Our results from 2017 to 2019 were similar to those found in studies by Clapp et al. [25] in the United States, where the authors found PHOs in the composition of 391 (9%) out of 4340 food products evaluated in 2012. It should be noted that at that time, the USA already had regulations in place that required the declaration of TFAs content on product labels [26]. In Brazil in 2010, over half of the 2327 products evaluated (50.5%; *n* = 1175) had one or more ingredients listed that were sources of i-TFAs [27]. This percentage decreased in subsequent years. In 2013, the aforementioned authors [28] found that 36% of 3176 food products available on the Brazilian market were potential sources of i-TFAs in the diet. In 2019, a further significant decrease was observed in the proportion of products in Brazil with labels containing both specific and non-specific references to i-TFAs sources [29]. In the overall evaluation of 11,434 products, these represented 4.1% and 14.6%, respectively.

A label evaluation of 8557 products in Slovenia in 2015 [30] showed that the proportion of products with PHOs ranged from 0.0% in the processed meat and derivatives category to 30.4% in vegetable cream substitutes. A similar evaluation conducted two years later [30], covering 14,072 products, showed that the proportion of products with PHOs ranged from 0.2% in processed meat and derivatives to 10.4% in cakes, muffins and pastry. In Australia, Huang et al. [31] found that only 131 products (0.5%) out of a total of 28,349 evaluated had ingredients listed that were sources of i-TFAs. Recent studies in Kenya and Nigeria [32] showed that products containing i-TFAs sources are still present in these countries’ markets at rates of 1.7% and 4.9%, respectively. The presented findings from studies around the world indicate that i-TFAs are still present in global food supplies, although a decline in the proportion of products containing PHOs in the total number of available products has been observed.

Assessment of product labels available on the Polish market between 2022 and 2023 revealed that none of the 779 analysed products contained PHOs in their composition. These products belonged to the same 40 groups and 12 food categories already assessed between 2017 and 2019. The findings indicate that Polish food manufacturers have complied with the legal requirements and confirm that Commission Regulation (EU) 2019/649 has effectively reduced the use of PHOs as the primary source of i-TFAs in food products in Poland. This is further corroborated by our analytical studies of TFAs content in food products conducted in Poland since 2017, as part of the National Health Programme task titled “Maintaining the e-Database of Trans Fatty Acid Isomer Content in Food” [33].

The findings of our study, similarly to the results of the aforementioned studies by other authors, indicate a positive global trend in reducing the presence of PHOs as a source of i-TFAs in food products. While voluntary measures taken by food manufacturers have contributed significantly to this outcome, the introduction of regulatory frameworks has been the key factor. Firstly, the obligation to declare the TFAs content on product labels, introduced in 2004 in Canada and subsequently adopted in the United States and several South American countries, has played a crucial role [34]. Secondly, the establishment of a legal limit for i-TFAs in food intended for the final consumers and food intended for supply to retail within the European Union was a critical measure [19]. A recent WHO report [35] revealed that 69 countries globally have enacted regulations to limit trans fat content in food. Among these, 53 countries, including Poland, have adopted best practice policies aimed at eliminating harmful trans fats. These policies include setting a legal limit for i-TFAs in food and/or banning the production or use of partially hydrogenated oils. Notably, in November 2023, Poland became one of the first five countries worldwide to receive the WHO’s Validation of Trans Fat Elimination certificate, recognising its successful elimination of i-TFAs from the national food supply [36].

It is important to emphasise that the reduction in the presence of PHOs in food products has directly contributed to a decrease in dietary TFAs intake. Wanders et al. [37] in a 2017, based on the review of 43 publications, reported that in nearly all countries included in the review, TFAs consumption had significantly declined over the past two decades. For instance, in Canada, within four years of implementing mandatory TFA labelling on products, dietary TFAs intake decreased by over 50% (7.7 g/day in 2004 versus 3.4 g/day in 2008). Similarly, Denmark saw an almost twofold reduction in TFAs intake from food between 1995 and 2012 (2.6 g/day in 1995–1996 compared to 1.5 g/day in 2012). During a comparable timeframe, Belgium experienced a more than fourfold reduction in TFAs intake, with levels decreasing from 4.1 g/day in 1995–1996 to 0.9 g/day in 2014. It appears that the introduction of relevant EU legislation in 2019 [19] has further reduced TFAs intake in diets. Our research [38], conducted during the enforcement of Commission Regulation (EU) 2019/649, revealed that the total TFAs content in hospital diets provided to maternity and gynaecology wards in Poland did not exceed 0.5% of dietary energy. This finding aligns with the WHO’s recommended guideline of no more than 1% of energy intake from TFAs [39].

The reduction in TFAs intake through the diet, which results from the elimination of PHOs in food production and consequently lowers the content of i-TFAs in food products, is also supported by the observed decrease in TFAs levels in breast milk over the last 20 years [16,40]. For example, in Brazil, over an eight-year period, the TFAs content in mature milk decreased by approximately 25% (2.19% wt/wt in 2008 vs. 1.65% wt/wt in 2016). Similarly, in Canada, between 1996 and 2011, there was a nearly fourfold reduction in the TFAs content in mature milk (7.19% wt/wt in 1996 vs. 1.9% wt/wt in 2011) [16].

In the current study, we also attempted to assess the type and extent of product reformulations implemented by producers to reduce and/or eliminate PHOs. For this purpose, in Study II (2022–2023), we evaluated the type of fat declared on the labels of 779 products, belonging to the same 40 groups from 12 food categories assessed in Study I (2017–2019). As previously mentioned, none of these products contained partially hydrogenated fats. However, it is important to note that nearly 40% (*n* = 309) of the products contained tropical oils and in over 80% of these products, palm oil was the sole source of fat. In the remaining 20% (*n* = 47), the products contained a mixture of palm and coconut oil or solely coconut oil. In comparison, in Study I, prior to the implementation of Commission Regulation (EU) 2019/649, we found tropical oils in the composition of 47% (*n* = 575) of the products assessed. In just over 60% (*n* = 470) of all the products evaluated during that period, plant oils other than tropical oils and animal fats were present.

Additionally, in Study II, we assessed the type of fat used in products that contained PHOs in Study I. Out of a total of 84 products with PHOs identified in Study I, 48 products (57%) with the same name and brand/manufacturer were found in retail sales. It appears that the remaining 43% of the products were withdrawn from production by the manufacturers. According to the manufacturer’s declaration on the label, none of the 48 products re-evaluated contained partially hydrogenated oils/fats. At the same time, the number of products containing tropical oils increased, as palm oil was the most commonly used replacement for PHOs. The use of tropical oils exclusively as PHOs substitutes applied to over half (*n* = 25; 52.1%) of the re-evaluated food products. A positive development was the replacement of PHOs with vegetable oils other than tropical oils, such as sunflower or rapeseed oil. However, this only applied to 33.3% of products. In the remaining products (14.6%), animal fats or a combination of tropical oils (mainly palm oil) and animal fats were used as substitutes for PHOs.

The reduction in the proportion of products containing PHOs and, consequently, the decrease in the content of i-TFAs in products, observed after the implementation of Commission Regulation (EU) 2019/649, is a very positive development from the perspective of preventing diet-related diseases, particularly cardiovascular diseases. However, it should be noted that tropical oils, especially palm oil, which contain high levels of saturated fatty acids, are used as substitutes for PHOs. In crude palm oil, approximately 50% of all fatty acids are saturated fatty acids [21]. Lee et al. [41], in a recently published study evaluating dietary trends in the United States since 1800, found that the increase in the consumption of processed foods was linked to the rising prevalence of chronic non-communicable diseases. At the same time, a decrease in the consumption of animal-derived saturated fats was noted, alongside an increase in the consumption of fats from vegetable oils. Therefore, it can be assumed that the current level of saturated fatty acid (SFAs) intake is less dependent on the content of animal fats in the diet and more on the content of tropical oils. According to the recommendations of the European Food Safety Authority (EFSA), SFAs intake should be as low as is possible within the context of a nutritionally adequate diet [42]. It appears that replacing PHOs with palm oil, a practice frequently used by manufacturers, may not mitigate the expected beneficial effects associated with the elimination of i-TFAs from food and diets, due to the significant amounts of saturated fatty acids introduced into the diet. This practice, therefore, may not constitute an effective tool in the prevention of diet-related diseases.

## 5. Limitations

A limitation of our study is the relatively small number of product labels assessed, particularly, in Study II (*n* = 779) after the implementation of Commission Regulation (EU) 2019/649. However, in Study I, we assessed the labels of food products from 12 different selected categories and 40 groups of food products, including products without PHOs, resulting in a relatively large sample size (*n* = 1224). It should be underlined that we have been conducting research on TFAs content in food for many years [33,43], thus possessing the necessary knowledge and experience in terms of the groups and categories of products that are sources of TFAs. We considered that since only 6.9% of all products in Study I contained PHOs, in Study II after the implementation of Commission Regulation (EU) 2019/649, we would assess the labels of no less than 50% of products from the same 12 categories and 40 groups of food products. Additionally, in Study II, we assessed the labels of the same products (brand, producer) where PHOs were present in Study I.

A second limitation was that the data from product labels were collected from one region and only from the largest national retail chains. We are aware that we did not cover the entire Polish market, including regional manufacturers. However, it should be emphasised that the retailers covered by the study accounted for over 50% of the total market share in Poland. Additionally, we used the same procedure for label analysis of food products available in retail outlets in 2017–2019 and 2022–2023 and the research was conducted in the same retail outlets.

Another possible limitation was the focus solely on packaged products. Consequently, we did not assess unpackaged food, such as various types of bakery products, including sweet bread, cakes and biscuits. However, this was part of the design of our study, which aimed to assess the type of fat used based on the information provided on the label. It is important to note that since 2017, as part of the National Health Program task, we have been conducting ongoing analytical research on TFAs content in randomly selected food products available on the Polish market, including unpackaged products, produced both by large manufacturers and local suppliers. The results of the studies are regularly published by updating the TFAs e-Base website [33].

## 6. Conclusions

The conducted studies indicate that the entry into force of the legal regulation specifying the legal limit for i-TFAs in food products intended for the final consumer [19] has effectively reduced the use of PHOs, which were previously the main source of i-TFAs in food. It is worth noting that, thanks to the voluntary actions of Polish food producers, even pre-introduction of Regulation 2019/649, products containing PHOs accounted for only 6.9% of all the products we tested. At the same time, it should be pointed out that tropical oils were a fat component in nearly 40% of the products, out of the total of 779 products whose labels we examined after the entry into force of Regulation (EU) 2019/649. Our results clearly indicate that tropical oils are most often used as substitutes for PHOs. This was also confirmed by the fact that in over 60% of the 48 products, which prior to the implementation of Regulation (EU) 2019/649 contained PHOs, tropical oils, primarily palm oil, were used as substitutes. It should be emphasised that tropical oils contain a significant proportion of saturated fatty acids among all fatty acids. Specifically, palm oil contains approximately 50% saturated fatty acids, while coconut oil contains about 90%. According to current recommendations [42], the intake of SFAs through diet should be as low as is possible within the context of a nutritionally adequate diet. This highlights the need for educational campaigns to increase awareness among both food producers and consumers. Additionally, to ensure consumers’ right to information, it is essential to conduct analytical studies and regularly publish up-to-date results. Our research provides valuable information to the public regarding the presence of specific ingredients that contribute to i-TFAs and tropical oils in commercially available products.

## Figures and Tables

**Table 1:** Keywords used to identify products containing PHOs and tropical oils.

Type of Fatty Acids/Fats	Searched Items *
Industrially produced trans fatty acids (i-TFAs)	partially hydrogenated/partially hardened vegetable oil/fat, partially hydrogenated/partially hardened animal fat, partially hydrogenated/partially hardened oil/fat
Tropical oils	palm oil/fat coconut oil/fat fully hydrogenated oil from palm seeds fully hydrogenated palm fat hydrogenated palm fat fully hydrogenated coconut oil non-hydrogenated palm fat vegetable fat from palm kernel vegetable fat from palm seeds vegetable fat from palm kernel seeds refined palm oil

* Note: In accordance with Regulation (EU) No. 1169/2011 of the European Parliament and of the Council of 25 October 2011 on the provision of food information to consumers, the indication of hydrogenated oil/fat (refined vegetable oils/fats and refined animal oils/fats) must be accompanied by the expression “fully hydrogenated/hardened” or “partially hydrogenated/hardened”.

**Table 2:** Products whose labels were assessed in Study I and Study II.

Food Category	Product Groups	Number of Products	
		Study I (2017–2019)	Study II (2022–2023)
I. Fast food products	- frozen pizza - pizza sauces	15	28
II. Food concentrates	- soup concentrates (ready to eat after cooking), - sauce concentrates (ready to eat after cooking), - instant soup concentrates, - instant noodle soups, - stock cubes, - salad dressings, - cake mixes, - cake frosting mixes, - instant dessert mixes	367	199
III. Confectionery products	- filled chocolates, - sesame halva, - sweet or flavoured bread spreads, - filled snack bars, - boxed sweets (e.g., pralines or assorted chocolate boxes), - marzipan	231	126
IV. Bakery products	- filled wafers, - cakes from chain bakeries, - packaged cakes, - biscuits (e.g., sponge biscuits, digestives, sandwich biscuits), - gingerbread, - biscuits from chain bakeries	148	87
V. Snack products such as chips and crisps	- potato crisps, - puffed potato snacks, - wheat and corn-based crisps/puffs	46	35
VI. Deli and culinary products	- croquettes (semi-finished products)	21	17
VII. Dairy products	- aged hard cheeses, - blue cheeses, - ice cream tubs (family packs)	153	94
VIII. Processed meat products	- canned meats, - canned meat pâtés, - meat sticks—kabanos	55	66
IX. Processed fish products	- fish pâtés	6	10
X. Processed vegetable products	- vegetable dips and hummus, - vegetable pâtés	13	10
XI. Mayonnaise, sauces, mustards	- mayonnaise, - hot meal sauces, - sandwich sauces	96	70
XII. Food for specific groups	- baby food jars (for infants and young children)	73	37
The total number of products whose labels were assessed in Study I and Study II		1224	779

**Table 3:** Number and percentage share (%) of food products available on the Polish market from 2017 to 2019 assessed in terms of the type of fat listed among the ingredients on the label, categorised by food groups.

Food Category	*N*	Type of Fat ^A,B^ [Number of Food Products (Percentage Share, %)]				
		Partially Hydrogenated Oils/Fats ^A^	Tropical Oils ^B^	Partially Hydrogenated Oils/Fats ^A^ and Tropical Oils ^B^	Hydrogenated Oils/Fats	Without Partially Hydrogenated Oils/Fats and Tropical Oils
I. Fast food products	15	0	5 (33%)	3 (20%)	0	7 (47%)
II. Food concentrates	367	33 (9%)	222 (60%)	7 (2%)	2 (1%)	103 (28%)
III. Confectionery products	231	11 (5%)	150 (65%)	7 (3%)	0	63 (27%)
IV. Bakery products	148	7 (5%)	108 (73%)	14 (9%)	4 (3%)	15 (10%)
V. Snack products such as chips and crisps	46	1 (2%)	26 (57%)	0	0	19 (41%)
VI. Dairy products	153	0	19 (12%)	0	0	134 (88%)
VII. Mayonnaise, sauces, mustards	96	0	2 (2%)	0	0	94 (98%)
VIII. Deli and culinary products	21	0	9 (43%)	0	0	12 (57%)
IX. Processed meat products	55	1 (2%)	1 (2%)	0	0	53 (96%)
X. Processed fish products	6	0	1 (17%)	0	0	5 (83%)
XI. Processed vegetable products	13	0	1 (8%)	0	0	12 (92%)
XII. Food for specific groups	73	0	0	0	0	73 (100%)
Total [number of food products (percentage share,%)	1224	53 (4.4%)	544 (44.4%)	31 (2.5%)	6 (0.5%)	590 (48.2%)

*N*—number of products. ^A^ The ingredient list of each product was searched using specific terms (see Table 1), including ‘partially hydrogenated palm oil’ which clearly indicate ingredients that are the main sources of industrially produced trans fatty acids (i-TFAs) in food. Non-specific terms, such as, for example, “Hydrogenated Vegetable Oil”, “Hydrogenated vegetable fat”, “Hydrogenated”, “Fully Hydrogenated”, “Vegetable Fat”, “Margarine”, “Vegetable Cream” which may or may not indicate i-TFAs-containing ingredients were excluded. ^B^ The ingredient list of each product was searched using specific terms (see Table 1) which clearly indicate products with tropical oils/fats present in the list of ingredients excluding partially hydrogenated palm oil and non-specific terms such as “Vegetable Oils”, “Vegetable fat” or “Hydrogenated Oils/Fats”.

**Table 4:** Number and percentage share (%) of food products containing partially hydrogenated oils/fats and/or tropical oils on the ingredient list, available on the Polish market in 2022–2023 and produced after the entry into force of Commission Regulation (EU) 2019/649.

Food Category	Type of Fat ^A^ [Number of Food Products (Percentage Share, %)]					
	*N*	with PHOs ^A^	with Tropical Oils			with Other Fats ^B^
			PO	CO	PO + CO	
I. Fast food products	28	0	11 (39.3%)			17
			9	2	0	
II. Food concentrates	199	0	129 (64.8%)			70
			118	1	10	
III. Confectionery products	126	0	83 (65.9%)			43
			76	2	5	
IV. Bakery products	87	0	55 (63.2%)			32
			43	0	12	
V. Snack products such as chips and crisps	35	0	7 (20.0%)			25
			6	1	0	
VI. Dairy products	94	0	24 (25.5%)			70
			0	22	2	
VII. Processed meat products	66	0	0			66
VIII. Mayonnaise, sauces, mustards	70	0	0			70
IX. Deli and culinary products	17	0	0			17
X. Processed fish products	10	0	0			10
XI. Processed vegetable products	10	0	0			10
XII. Food for specific group	37	0	0			37
Total [number of food products (percentage share, %)]	779	0	252 (32.4%)	28 (3.6%)	29 (3.7%)	470 (60.3%)

*N*—number of products; PHOs—Partially Hydrogenated Oils/Fats; PO—Palm Oils, CO—Coconut Oils; PO + CO—Palm Oils and Coconut Oils. ^A^—the ingredient list of each product was searched using specific terms (see Table 1) including partially hydrogenated tropical palm and coconut oil. ^B^—fats other than PHOs and tropical oils, e.g., vegetable oils (sunflower, rapeseed), animal fats.

**Table 5:** Number and percentage share (%) of food products whose composition was reformulated after the implementation of Regulation 649/2019 by replacing PHOs with other types of fats.

Food Category	*N*	Types of Fats Used to Replace PHOs [Number (Percentage Share, %) of Food Products]			
		Tropical Oils ^A^	Tropical Oils ^A^ and Animal Fats ^B^	Vegetable Oils ^C^	Animal Fats ^B^
Food concentrates	23	11 (48%)	3 (13%)	7 (30%)	2 (9%)
Confectionery products	10	3 (30%)	1 (10%)	5 (50%)	1 (10%)
Bakery products	13	9 (69%)	0	4 (31%)	0
Snack products such as chips, crisps	1	1 (100%)	0	0	0
Processed meat and derivatives	1	1 (100%)	0	0	0
Total [number (percentage share, %) of food products]	48	25 (52.1%)	4 (8.3%)	16 (33.3%)	3 (6.3%)

*N*—number of food products. ^A^ Palm oil and/or coconut oil: The ingredient list of each product was searched using specific terms (see Table 1). ^B^ Fats derived from animal fat tissue and/or milk: beef extract, milk fat. ^C^ Vegetable oils other than tropical oils (palm and coconut oils): rapeseed oil, sunflower oil. Note: Based on the results of the comparison of the ingredient lists of the examined food products, four types of substitutes for partially hydrogenated oils/fats were identified. The examined food products (*n* = 48) were classified into one of these four groups.

## Data Availability

The original contributions presented in the study are included in the article, further inquiries can be directed to the corresponding authors.

## Abbreviations

The following abbreviations are used in this manuscript:

TFAs	trans fatty acids
i-TFAs	industrially produced trans fatty acids
r-TFAs	ruminant trans fatty acids
SFAs	saturated fatty acids
PHOs	partially hydrogenated oils
WHO	World Health Organization
EFSA	European Food Safety Authority
